# High-percentage new energy distribution network line loss frequency division prediction based on wavelet transform and BIGRU-LSTM

**DOI:** 10.1371/journal.pone.0308940

**Published:** 2024-08-19

**Authors:** Xiangming Wu, Nan Song, Jifeng Liang, Ye Lv, Zitian Wang, Lijun Yang

**Affiliations:** 1 State Grid Hebei Electric Power Co., Ltd., Shijiazhuang City, Hebei Province, China; 2 Electric Power Science Research Institute, State Grid Hebei Electric Power Co., Ltd., Shijiazhuang City, Hebei Province, China; 3 Hebei Key Laboratory of Power Electronics Energy Conservation and Transmission Control (Yanshan University), Qinhuangdao City, Hebei Province, China; Wuhan University, CHINA

## Abstract

The access of new energy improves the flexibility of distribution network operation, but also leads to more complex mechanism of line loss. Therefore, starting from the nonlinear, fluctuating and multi-scale characteristics of line loss data, and based on the idea of decomposition prediction, this paper proposes a new method of line loss frequency division prediction based on wavelet transform and BIGRU-LSTM (Bidirectional Gated Recurrent Unit-Long Short Term Memory Network).Firstly, the grey relation analysis and the improved NARMA (Nonlinear Autoregressive Moving Average) correlation analysis method are used to extract the non-temporal and temporal influencing factors of line loss, and the corresponding feature data set is constructed. Then, the historical line loss data is decomposed into physical signals of different frequency bands by using wavelet transform, and the multi-dimensional input data of the prediction network is formed with the above characteristic data set. Finally, the BIGRU-LSTM prediction network is built to realize the probabilistic prediction of high-frequency and low-frequency components of line loss. The effectiveness and applicability of the method proposed in this paper were verified through numerical simulation. By dividing the line loss data into different frequency bands for frequency prediction, the mapping relationship between different line loss components and influencing factors was accurately matched, thereby improving the prediction accuracy.

## 1. Introduction

Since 2020, the central government has repeatedly reiterated the energy and power development goals of "building a new type of power system with new energy as the main body", "by 2030, the proportion of non-fossil energy in primary energy consumption will reach about 25%, and the total installed capacity of wind and solar power will reach over 1.2 billion kilowatts", and has put forward clear requirements for the green and low-carbon transformation of the power sector. However, the high proportion of grid connected new energy generation not only changes the structure and operation status of the distribution network, but also makes the mechanisms and related factors affecting line loss more complex and diverse, bringing new challenges to line loss prediction and management.

In the calculation of theoretical line loss of distribution network, the loss factor method and loss coefficient method based on engineering experience have appeared earlier. Due to the lack of theoretical basis, when the line current is negative or fluctuates greatly, the estimated line loss is quite different from the actual line loss. Other commonly used equivalent resistance method, root mean square current method and average current method also have the above problems. [1] gives a line loss evaluation method based on hybrid clustering analysis, which can obtain the probability distribution curve of line loss with less calculation scale. [2] proposed an engineering calculation method of theoretical line loss rate benchmark value considering line attributes, operation parameters and management factors. In [3], the load current curve is obtained by polynomial approximation and ninth order fitting. The above method is simple and intuitive, and can get more reasonable results for specific types of traditional distribution networks, However, with the access of new energy, the requirements for the flexible operation ability of the distribution network have been greatly improved, and the time-varying power flow of the distribution network has become increasingly prominent. The traditional line loss calculation method is faced with the problems of high modeling difficulty, large amount of calculation and poor calculation accuracy, which is difficult to be widely applied in the daily line loss management.

With the promotion of smart grid and digital grid, and the rapid development of big data processing technology, data-driven model prediction and decision-making methods are also gradually showing advantages in power grid. In terms of load forecasting and power forecasting, [4] based on personalized federated learning of long short-term memory network to predict photovoltaic output and load power. [5] realizes ultra-short-term load forecasting based on Prophet and double multi head self-attention time convolution network. At the same time, there are also relevant applications in the medium and long-term prediction of line loss. [6] improves the deep belief network model and proposes the Cycle-DBN-A model, which is used for prediction. [7] uses the method based on cross validation and gradient boosting decision tree to predict transmission line losses. In order to improve the prediction accuracy, [8] applies particle swarm optimization algorithm to extreme learning machine, and proposes a line loss prediction method based on PSO and elm. In the research of short-term line loss prediction, [9] uses Kmeans-LightGBM realize the short-term line loss prediction of low-voltage distribution network. Data driven method can effectively avoid the related problems of prediction based on mechanism modeling. It can not only express the regression characteristics of static data, but also reflect the complex correlation between line loss and electrical, environmental and other factors, as well as the dynamic time series law of historical data. It is becoming a hot method at present. However, the above-mentioned forecasting method only focuses on the historical data of load or line loss, does not explore the rich multi-scale information contained in the historical data, and does not mine all the key features of the original data, resulting in certain limitations of the forecasting results, and the accuracy needs to be further improved.

Aiming at the problem that the existing prediction models do not fully mine all the information contained in the original data, [10] proposes a time series prediction model based on EMD (Empirical Mode Decomposition), which is suitable for predicting time series with large amount of data and high volatility. EMD decomposes the original series into a series of subsequences, uses the appropriate prediction model for training, and then integrates all subsequences into the final prediction result. [11] uses VMD (Variational Mode Decomposition) to decompose the original sequence into multiple modal components, and divides them into high-frequency and medium low-frequency components, then uses different prediction models to predict, and finally adds the prediction results to obtain the final prediction result. [12] proposes a prediction hybrid model, which first uses adaptive white noise to complete EMD decomposition, decomposes the original sequence into high-frequency and low-frequency components, then further decomposes the high-frequency components using VMD method, and integrates the sub sequences obtained from the two decompositions according to the sample entropy. Finally, the fitting values of different frequency components in the training period are used as the input of the prediction model to obtain the final prediction results. This method of frequency division prediction can better capture the information of different frequency bands in the original data, enhance the multi-scale analysis ability of the prediction model, and obtain more accurate results. However, the above method does not combine the data of each frequency band with the influencing factors, and ignores the role of the influencing factors in the prediction model. At the same time, the above deterministic prediction method has certain limitations in expressing the fluctuation and uncertainty of line loss in new energy distribution network.

The source load of new energy power system has significant uncertainty, which is an important issue in its operation and planning. At present, probability method is mainly used to characterize the uncertainty of resource output or parameters in various application scenarios considering uncertainty. [13] realizes probabilistic load forecasting by combining the point prediction of residual error and conditional distribution model. [14] characterized the uncertainty caused by the high penetration of renewable energy with confidence. [15] deals with the uncertainty of load and equipment parameters through interval analysis method. [16] proposed a new nonparametric bootstrap method, which provides a clearer and better calibrated confidence interval for the estimation uncertainty of network degree distribution function. With the wide application of neural network, the probabilistic representation method based on neural network to express the uncertainty of objects has also been studied. [17] combines the fuzzy theory with the principle of neural network, and obtains an uncertain data flow modeling algorithm through radial basis function neural network. [18] proposed a fuzzy cerebellar model neural network structure for uncertain nonlinear systems, which effectively solved the uncertainty problem of nonlinear systems. It can be seen that a variety of modeling methods representing uncertainty have been widely used in many fields, and their combination with neural networks will bring new solutions to the modeling of uncertain problems, so as to better break through the limitations of deterministic modeling. At present, there are few literatures using neural network to represent the probability in the field of line loss, so it is necessary to carry out in-depth exploration in this area.

To sum up, the concept and technical method of load and power prediction and frequency division prediction are used for reference to avoid the difficulty in training the data-driven prediction model caused by the coupling of relationship characteristics and uncertainty distribution characteristics when the actual line loss changes, and fully express the complex coupling relationship between multiple influencing factors of line loss and the mapping relationship between line loss data, This paper proposes a frequency division prediction framework of BIGRU-LSTM high proportion new energy distribution network line loss based on wavelet transform. The main contents are as follows: (1) based on the electrical, environmental and other related factors that affect the line loss of the new energy distribution network, the grey relation analysis and the improved NARMA correlation degree analysis method are used to extract the non-sequential and sequential key characteristic parameters that affect the line loss generation, and the corresponding multidimensional characteristic data sets of line loss impact are constructed respectively. (2) Considering that the line loss of the distribution network with a high proportion of new energy access has the characteristics of strong volatility and trend change, the complex historical line loss data is divided into physical signals with different volatility and time scales in several different frequency bands by using wavelet transform, so as to map the main influence relationship of the data in different frequency bands, reduce the complexity of the prediction model and improve the prediction accuracy and adaptability of the new energy distribution network, The multi-dimensional input data of the prediction network is composed of the non-sequential and sequential key characteristic parameters extracted above. (3) Build the BIGRU-LSTM dual channel line loss prediction network, respectively match the mapping relationship between the time-series and non time-series key characteristic parameters and the high-frequency and low-frequency data components of line loss, so as to realize the deterministic and probabilistic frequency division prediction of the low-frequency and high-frequency decomposition components of line loss (4) output the line loss prediction results by superimposing the two channel prediction results through inverse wavelet transform. (5) Based on the example simulation, it is verified that the proposed method improves the multi-scale analysis ability of the prediction model, and can further improve the prediction accuracy when applied to the line loss prediction of high proportion of new energy distribution network.

## 2. Fundamentals of line loss prediction method based on wavelet transform and BIGRU-LSTM

**Fig 1:**
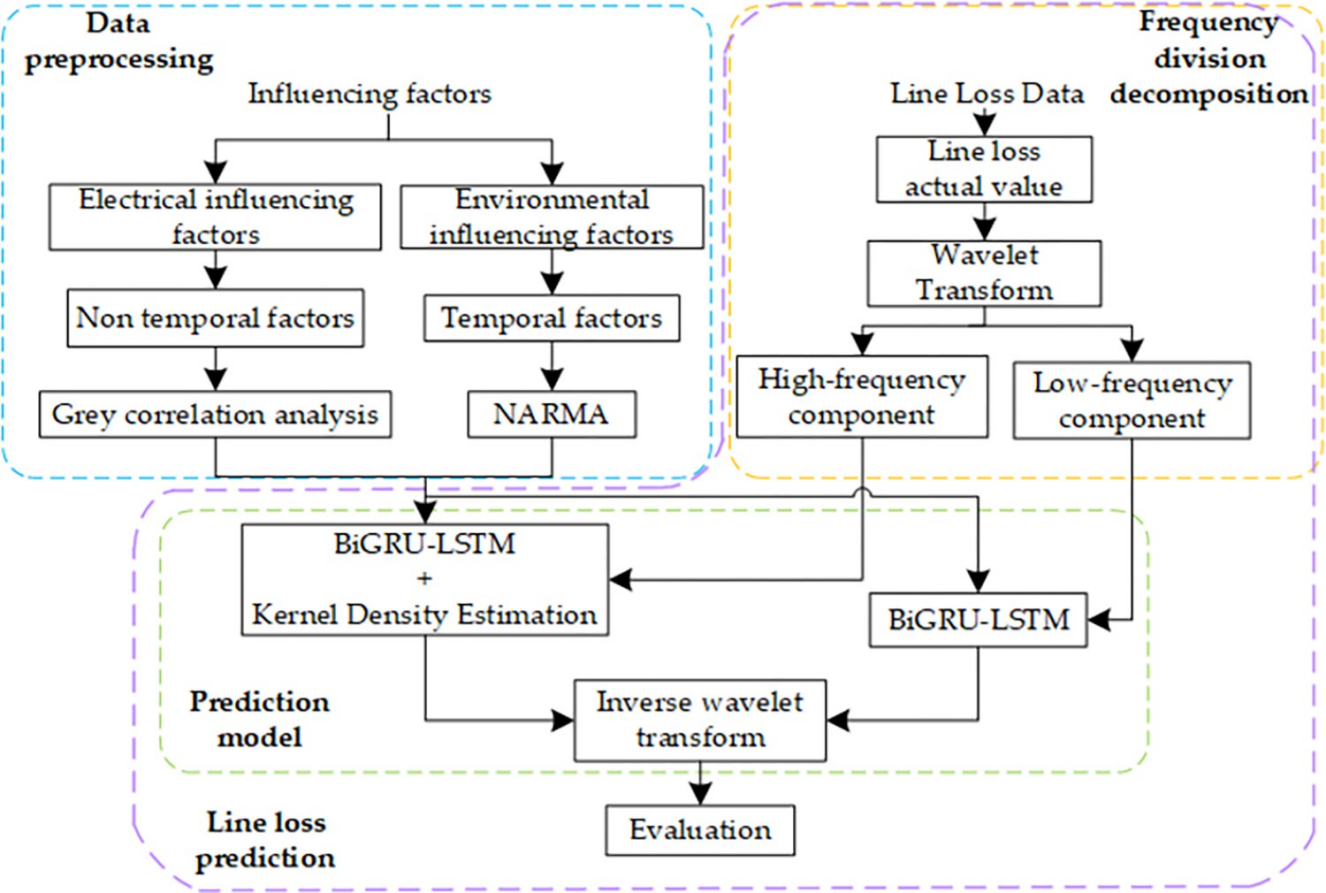
The basic architecture of line loss prediction method.

The basic flow of the line loss prediction method based on wavelet transform and BIGRU-LSTM is shown in Fig 1.

Among them, the specific steps and methods of the data preprocessing module are detailed in Section 2 of this paper. The specific contents of the line loss prediction framework composed of the frequency decomposition module and the prediction model module are detailed in Section 3 of this paper. The specific processes are as follows:

1. For the historical impact factors consisting of two parts, electrical factors and environmental factors, divide the two parts into non-timing factors and timing factors according to whether they have timing information or not; extract the key features of non-timing and timing, use grey relation analysis and NARMA model to extract the key features of extracting non-timing factors and timing factors respectively, and construct a multidimensional input data set of impact factors.
2. The BIGRU-LSTM line loss frequency prediction framework based on wavelet transform algorithm is proposed. Firstly, the historical line loss data is decomposed into high-frequency components and low-frequency components by wavelet transform. Second, the BIGRU-LSTM model is constructed, and for the low-frequency component, the deterministic prediction method based on BIGRU-LSTM is used, and for the high-frequency part which is more fluctuating, the probabilistic prediction model combining kernel density estimation and BIGRU-LSTM is constructed.
3. After obtaining the prediction interval of the high-frequency component of line loss through probabilistic prediction, extract the upper and lower bounds of the prediction interval and reconstruct them with the low-frequency deterministic prediction value using inverse wavelet transform to obtain the prediction results of line loss of new energy distribution network with a high proportion.
4. Evaluation of line loss prediction effect. Probabilistic indicators are used for performance evaluation, while deterministic indicators are used to assist in illustrating the prediction effect.

## 3. Extraction of characterizing factors

There are many factors affecting line loss in new energy distribution networks, and in order to reduce the complexity of model training, it is necessary to screen and eliminate the weakly correlated factors of line loss and extract the key factors affecting line loss. The correlation analysis method applies to both time series and non-time series data. Considering the different time nature, dynamic relationship, and influence mechanism between the non-time series and time series factors, it is proposed to take different methods to deal with the non-time series factors and time series factors.

**Fig 2:**
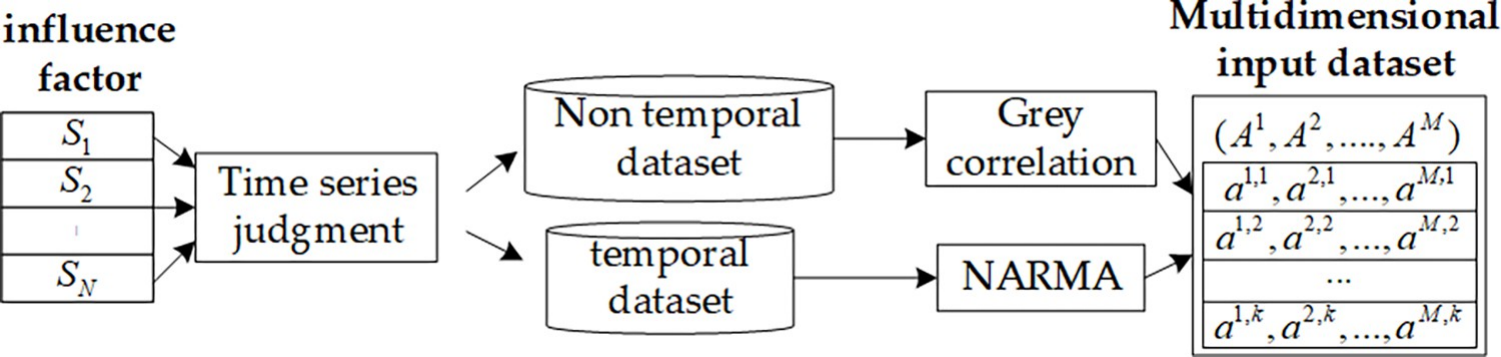
Data preprocessing framework of line loss influencing factors.

In practice, the timing factors changes all the time, while the non-timing factors do not change since they are determined, such as line type, length, transformer capacity, etc. When only studying the relationship between timing factors, the non-timing factors can be regarded as constants. At the same time, because the impact of line loss generated by changes in non-chronological factors is much larger than the impact of time-ordered changes in factors, the impact of time-ordered factors is not taken into account in the study of the importance of the relationship between non-time-ordered influencing factors. The preprocessing framework of line loss influencing factors data is shown in Fig 2.

### 3.1. Extraction of non-temporal influencing factors of line loss based on grey relation analysis

The values of non-temporal factors are unchanged in a certain time range, such as line type and material. In this paper, grey relation analysis is used to describe the complex static relationships between non-temporal factors. Grey relation analysis is suitable for dealing with ambiguous and incomplete information, and does not need to assume the functional relationship between the factors in advance, which has certain advantages in the extraction of non-timing influencing factors [19].

In the high proportion new energy distribution network, the non-timing influencing factors mainly include the total capacity of the public transformer, line length, line type (including material, cross-sectional area, insulation performance and other parameter information), reactive power compensation device capacity, etc. The non-timing influencing factors are summarized as follows. Each non-timing influencing factor is processed into the form of a column vector separately, and one influencing factor corresponds to one column of data, as shown in Eq (1).

$$X_{1}=[\begin{array}{l} x_{1}(1)\\ x_{1}(2)\\ \cdots \\ x_{1}(n) \end{array}],X_{2}=[\begin{array}{l} x_{2}(1)\\ x_{2}(2)\\ \cdots \\ x_{2}(n) \end{array}],\ldots ,X_{i}=[\begin{array}{l} x_{i}(1)\\ x_{i}(2)\\ \cdots \\ x_{i}(n) \end{array}],\ldots$$ (1)

where *X*_*i*_(*i* = 1,2,…) denotes the non-temporal nonlinear influences, *x*_*i*_(*i* = 1,2,…,*n*) denotes each element in the influences, and *n* denotes the total sample size.

The expressions for grey relation analysis are Eqs (2)-(3). For *ρ*∈(0,1),

$$\xi (x_{i}(k),c_{j}(k))=\frac{\underset{j}{\min}\;\underset{k}{\min}|x_{i}(k)-c_{j}(k)|+\rho \cdot \underset{j}{max}\;\underset{k}{max}|x_{i}(k)-c_{j}(k)|}{\left|x_{i}(k)-c_{j}(k)|+\rho \cdot \underset{j}{max}\;\underset{k}{max}|x_{i}(k)-c_{j}(k)\right|}$$ (2)

$$\xi (X_{i},C_{j})=\frac{1}{n}\sum_{k=1}^{n}\xi (x_{i}(k),c_{j}(k)),\;j=1,2,\ldots ,m$$ (3)

Where *ρ* is the weight of the maximum and minimum difference, *ξ*(*x*_*i*_(*k*), *c*_*j*_(*k*)) is the grey relation coefficient, and *ξ*(*X*_*i*_, *C*_*j*_) is the grey relation degree.

If a column has a high degree of correlation with the main sequence, it means that this influence factor has a high degree of overlap with the influence factor represented by the main sequence, that is, the deletion of this column has less influence on the whole influence factor system, and from the point of view of the arithmetic cost, it is possible to remove this kind of high overlap factor to realize the influence factor data dimensionality reduction. Finally, we get the influence factor data set which is not strong in relevance, but can effectively characterize the multi-dimensional features of the new energy distribution network, as shown in Eq (4).

$$C_{1}=[\begin{array}{l} c_{1}(1)\\ c_{1}(2)\\ \ldots \\ c_{1}(n) \end{array}],\ldots ,C_{j}=[\begin{array}{l} c_{j}(1)\\ c_{j}(2)\\ \ldots \\ c_{j}(n) \end{array}],\ldots ,C_{m}=[\begin{array}{l} c_{m}(1)\\ c_{m}(2)\\ \ldots \\ c_{m}(n) \end{array}]$$ (4)

where *C*_*j*_(*j* = 1,2,…,*m*) denotes the non-temporal nonlinear key influences, *c*_*i*_(*i* = 1,2,…,*n*) denotes each element in the key influences, and *n* denotes the total sample size.

### 3.2. Extraction of line loss timing influencing factors based on NARMA modeling

Time sequence factors refer to the factors that change with time, such as lighting, temperature, load and other factors that reflect the state change of comprehensive line loss. There is a dynamic time-series dependency between the factors. In this paper, the non-linear autoregressive moving average model with RNN as the kernel function is used to extract the influencing factors of line loss time-series. NARMA adopts a recursive network structure, which can extract the internal nonlinear dynamic relationship of the time-series system [20]. The model expressed in the form of past, current and future system parameters is shown in Eq (5).


$$y_{\left(k+d\right)}=f[y_{\left(t\right)},y_{\left(t-1\right)},\cdots ,y_{\left(t-n+1\right)},u_{\left(t\right)},u_{\left(t-1\right)},\cdots \cdot ,u_{\left(t-n+1\right)}]$$ (5)

**Fig 3:**
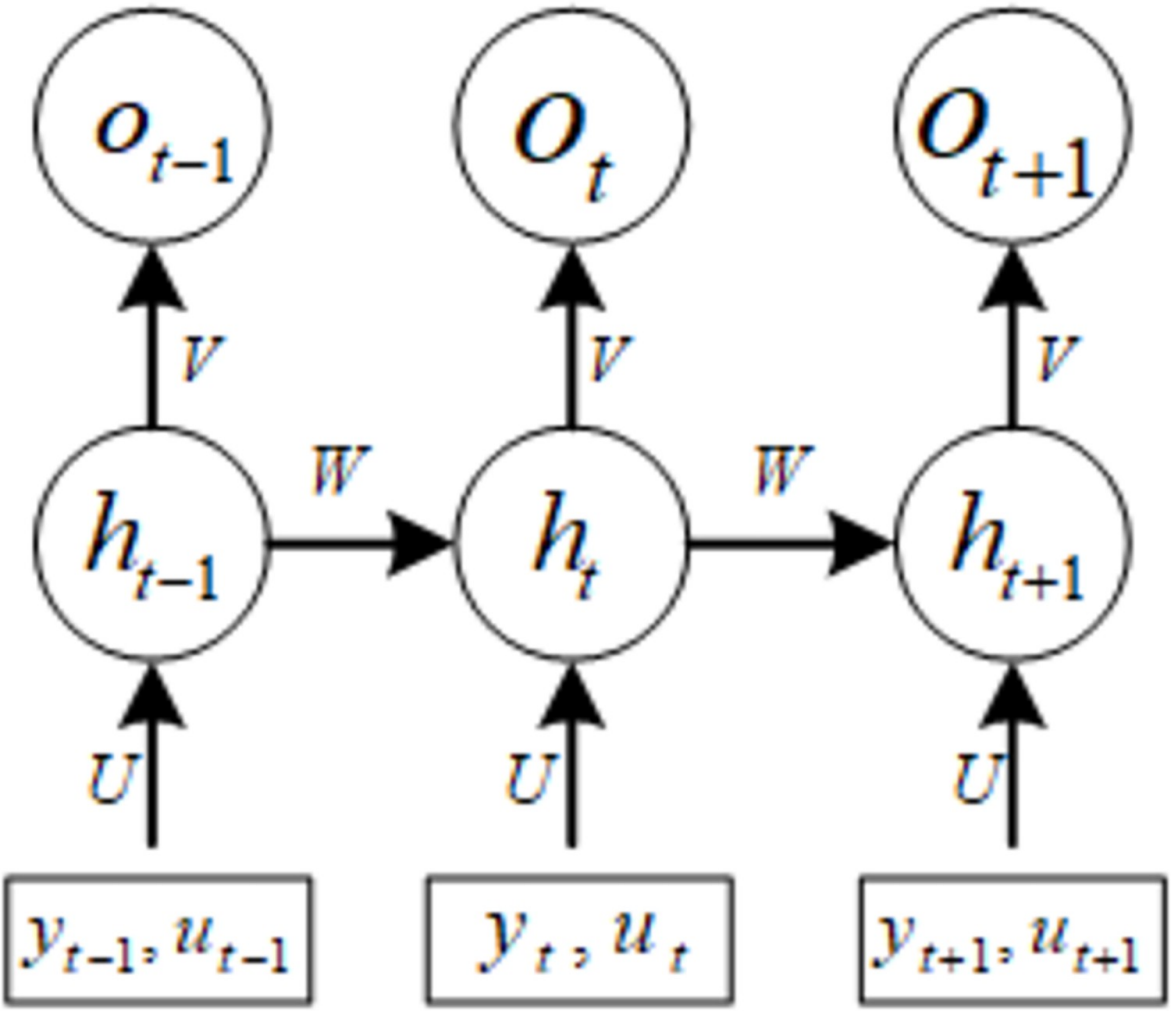
NARMA model structure diagram.

As shown in Fig 3, *y*_(*t*)_ and *u*_(*t*)_ are the output and input of the system respectively, *o*_(*t*)_ is the correlation degree of the influencing factors of the final output, and D is a nonlinear function. NARMA model uses past output and input values to fit the dynamic characteristics of the system, capture the nonlinear relationship between output and input, and extract the weight of influencing factors.

To improve the efficiency of the extraction process of key timing influencing factors, and at the same time to facilitate the subsequent data processing, this paper uses a neural network as *f*[*] in Eq (5), so that the weights of the neural network can be interpreted as the correlation between each input variable and the line loss, and it can be assumed that the influencing factors with high weights represent the key influencing factors at the level of the timing of the line loss.

Through this correlation analysis method based on NARMA basic framework and RNN neural network as a nonlinear function, it can not only capture the time-series dependence of influencing factors through the structure of circulating neurons, but also capture the nonlinear coupling relationship between influencing factors and line loss through the nonlinear autoregressive moving average method, which enhances the modeling ability of NARMA model for time series data and improves the extraction accuracy of influencing factors of line loss time series, so as to lay a solid foundation for the prediction process based on wavelet transform and predict more accurately.

In the high proportion of new energy distribution network, the time series factor data mainly includes active power supply, reactive power supply, temperature, wind speed, humidity, rainfall, etc. Different from the grey correlation method, the RNN recurrent neural network, as a nonlinear function of NARMA, can store the time series factors in the influencing factor data. The NARMA based on Eq (5) requires setting the target quantity. In this paper, various time series influencing factors are input as input data, and historical line loss data are input as target quantity, and the RNN network is trained to further extract network weights to determine the importance ranking of the time series influencing factors of line loss.

## 4. Line loss prediction framework for high percentage new energy distribution networks

Firstly, based on the historical line loss data, the distribution network loss caused by a high proportion of new energy (solar, wind) access is divided into basic loss and fluctuating loss, and the wavelet transform is used to decompose the line loss data into low-frequency (basic) and high-frequency (fluctuating) parts. Then based on the BIGRU-LSTM network realize the prediction of low-frequency line loss, using the combination of BIGRU-LSTM network and Gaussian kernel density estimation to realize the probabilistic interval prediction of high-frequency line loss.

### 4.1. Extraction of line loss timing influencing factors based on NARMA modeling

Because new energy power generation is closely related to environmental and meteorological conditions, its output has typical characteristics of volatility and randomness. Therefore, the new energy grid adopts a more flexible load supply mode to absorb new energy and realize the safe, stable and economic operation of the grid, resulting in the fluctuation characteristics of line loss in different frequency bands. Therefore, this paper considers using wavelet transform to decompose the line loss data, in order to better analyze the complexity characteristics of the line loss waveform in the background of new energy distribution network, and lay the foundation for the subsequent frequency division prediction. Wavelet transform can separate the high-frequency quantities representing volatility from the low-frequency quantities representing base loss from the perspective of frequency, while retaining the time information [21]. The expression of wavelet transform is shown in Eq (6):

$$\mathrm{WT}(a,\tau )=\frac{1}{\sqrt{a}}\int_{-\infty }^{\infty }f(t)\cdot \psi (\frac{t-\tau }{a})dt$$ (6)

where *a* is called the scale factor, which is used to deflate the fundamental wavelet *ψ*(*t*), *τ* reflects the displacement, which can be positive or negative, *a* and *τ* are continuous variables.

Based on the crossover frequency line loss prediction results, it is necessary to use wavelet inverse transform to reduce the decomposed waveform to a whole again to express the actual line loss prediction.

For any scale *J*:

$$f(t)=\sum_{k=-\infty }^{\infty }C_{J,k}\phi_{J,K}(t)+\sum_{j=1}^{J}\sum_{k=-\infty }^{\infty }d_{j,k}\psi_{j,k}(t)$$ (7)

If the wavelet basis function *ψ*_*j*,*k*_(*t*) is an orthogonal basis, and *J*→∞, the function can be represented by the detail part, i.e., there is an inverse transformation:

$$f(t)=\sum_{j=1}^{J}\sum_{k=-\infty }^{\infty }d_{j,k}\psi_{j,k}(t)$$ (8)

where $C_{J,K}=\int_{-\infty }^{\infty }f(t)\phi_{j,k}(t)dt$ is the scale factor associated with *f*(*t*) and *j*; $d_{j,k}=\int_{-\infty }^{\infty }f(t)\psi_{j,k}(t)dt$ denotes the wavelet coefficients of the function *f*(*t*).

### 4.2. Extraction of line loss timing influencing factors based on NARMA modeling

Based on the high and low frequency components and feature component data of the aforementioned line loss decomposition, this paper constructs a crossover prediction model based on the BIGRU-LSTM network, which is a composite neural network composed of BIGRU and LSTM. Where the BIGRU hidden layer state output is Eq (9):

$$\{\begin{array}{l} \vec{h_{t}}=GRU(x_{t},\vec{h_{t-1}})\\ \overset{\leftarrow }{h_{t}}=GRU(x_{t},\overset{\leftarrow }{h_{t-1}})\\ h_{t}=w_{t}\vec{h_{t}}+v_{t}\overset{\leftarrow }{h_{t}}+b_{t} \end{array}$$ (9)

where *w*_*t*_, *v*_*t*_ denote the weights corresponding to the forward hermit state $\vec{h_{t}}$ and the reverse hermit state $\vec{h_{t}}$ corresponding to the BIGRU at time *t*, and *b*_*t*_ denotes the bias corresponding to the hermit state at time *t*.

**Fig 4:**
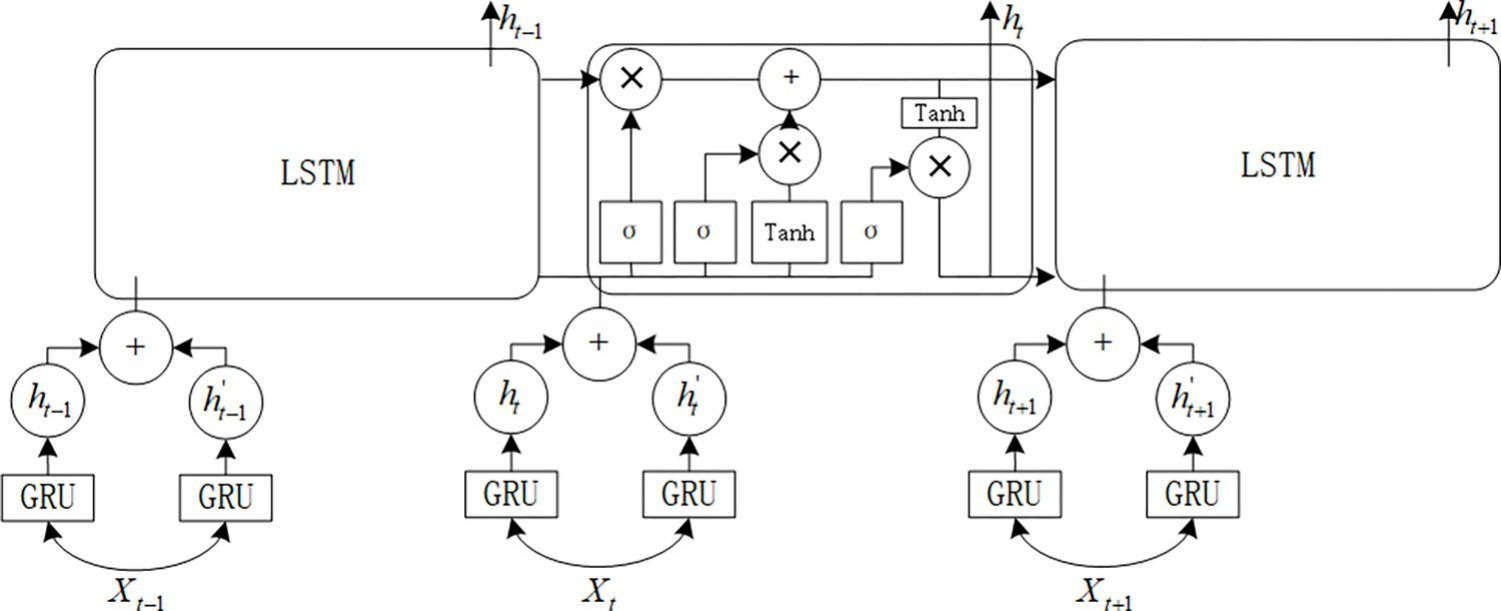
BIGRU-LSTM line loss prediction model structure.

The LSTM structure with forgetting gate, input gate and output gate expressions are shown in Eqs (10)–(12):

$$f_{t}=\sigma (W_{f}\cdot [h_{t-1},x_{t}]+b_{f})$$ (10)

$$\tilde{C_{t}}=\tanh\left(W_{C}\cdot [h_{t-1},x_{t}]+b_{C}\right)$$ (11)

$$o_{t}=\sigma (W_{o}\cdot [{h_{t}}_{-1},x_{t}]+b_{o})$$ (12)

where the weight coefficients of *x*_*t*_, *h*_*t*−1_ are *W*_*f*_、*W*_*C*_、*W*_*o*_ respectively; and the bias vectors are *b*_*f*_、*b*_*C*_、*b*_*o*_ respectively. Synthesizing the structural features of BIGRU and LSTM, the structure of BIGRU-LSTM is shown in Fig 4.

In Fig 4., BIGRU captures the temporal order and reverse temporal order information of the input data sequence through a bi-directional structure, i.e., it captures the effect of past information on the future as well as the effect of future information on the past. In this way, the BIGRU model can take into account both historical and future information at each point in time to better understand the patterns of the sequence data. LSTM, on the other hand, can selectively "remember" or "forget" the past information by utilizing its unique gating mechanism, thus effectively capturing the temporal dependence of the past information on future results [22]. The proposed BIGRU-LSTM line loss prediction model is expected to be able to utilize the advantages of the two separate model structures and provide two channels to predict the high and low frequency line loss components, to more accurately predict the trend of line loss and express the distribution of line loss.

### 4.3. Gaussian kernel density estimation

Due to the existence of a large number of high-frequency components in the high percentage of new energy distribution network line loss datasets, the traditional deterministic prediction methods cannot realize the effective quantification of the high-frequency components. Therefore, a probabilistic prediction method based on Gaussian kernel density estimation is introduced to model and quantify the high-frequency components.

Gaussian kernel density estimation is a nonparametric method that uses a Gaussian kernel function to estimate the sample distribution and does not require a priori knowledge as a basis, allowing the distribution to be fitted based solely on the characteristics and properties of the data. Its density estimation function is shown in Eqs (13)–(14).

$$\sigma_{Ke\mathrm{rn}el}(\xi_{0})=\frac{1}{N}\sum_{i=1}^{N}Kernel(\xi_{i}-\xi_{0};\mu )$$ (13)

$$Kernel(\xi_{i}-\xi_{0};\mu )=\frac{1}{\sqrt{2\pi }\mu }exp[-\frac{1}{2}{\left(\frac{\xi_{i}-\xi_{0}}{\mu }\right)}^{2}]$$ (14)

where *σ*_*Kernel*_ is the kernel density at sample *ξ*_0_; *Kernel*(*) is the kernel function; *ξ*_*i*_ is the sample *i*; and *μ* is the hyperparameter of the Gaussian kernel density estimation, representing the bandwidth of the kernel density estimation. If *μ* is set large, the corresponding density distribution is relatively smooth and vice versa.

### 4.4. Predictive accuracy indicators

To evaluate the effectiveness of the BIGRU-LSTM model in the work of online loss prediction, the probabilistic prediction results, the prediction interval coverage, the prediction interval width, and the combined weighted scores of the two are used as the evaluation indexes, as shown in Eqs (15)–(17).


$$\delta_{cov}=\frac{1}{N}\sum_{t=1}^{N}\psi \{{\hat{l}}_{t}\in I_{\beta }(l_{t})\}$$ (15)

$$\chi_{cov}=\frac{1}{N}\sum_{t=1}^{N}\Delta [I_{\beta }(l_{t})]$$ (16)

$$Score=W_{1}\delta_{\mathrm{cov}}-W_{2}\chi_{\mathrm{cov}}$$ (17)

Where *I*_*β*_(*l*_*t*_) refers to the confidence interval at the confidence level of *β*; *Δ*[*I*_*β*_(*l*_*t*_)] refers to the width of the prediction interval at the confidence level of *β*; *ψ*{*} is the indicative function, whose value is 1 when the condition is established, and 0 otherwise; *W*_1_ and *W*_2_ denote the weighting coefficients. Under the same prediction interval coverage, the smaller the prediction interval width, the higher the prediction accuracy of the model. Meanwhile, the larger *Score* is, the higher is the prediction accuracy of the model.

In the deterministic prediction index, the middle value of the prediction interval can be taken as the reference for the prediction performance of the model. Select the mean absolute error, mean absolute percentage error, root mean square error, R-square and other indicators to analyze the difference between the line loss prediction and the actual value, as shown in Eqs (18)–(21). The smaller the value of the first three indicators, and the closer the value of R2 is to 1, the higher the prediction accuracy.


$$MAE=\frac{1}{n}\cdot \sum \left|y_{i}-\hat{y_{i}}\right|$$ (18)

$$MAPE=\frac{1}{n}\cdot \sum \left(\left|\left(y_{i}-\hat{y_{i}}\right)/y_{i}\right|\right)\%$$ (19)

$$RMSE=\sqrt{\frac{1}{n}\cdot \sum {\left(y_{i}-\hat{y_{i}}\right)}^{2}}$$ (20)

$$R^{2}=1-(\sum {\left(y_{i}-\hat{y_{i}}\right)}^{2}/\sum {\left(y_{i}-\bar{y_{i}}\right)}^{2})$$ (21)

Where *n* is the total number of samples, ${\hat{y}}_{i}$ is the predicted result, and *y*_*i*_ is the real result.

## 5. Simulations

This paper selects the line loss data of a regional distribution network in China. The sampling interval is 1 hour, and the data set contains 3000 hours of data. The proportion of new energy access in this area is 20%, and the weather data are the direct solar radiation intensity, temperature, humidity, wind speed, atmospheric pressure and wind direction collected by the corresponding weather station. Since the size of the training data set is an important factor that directly affects the prediction accuracy, based on the strategy of Singla’s data set division in reference [23] and previous experience, this paper classifies our data set in training–testing ratio of 80%: 20%.

**Fig 5:**
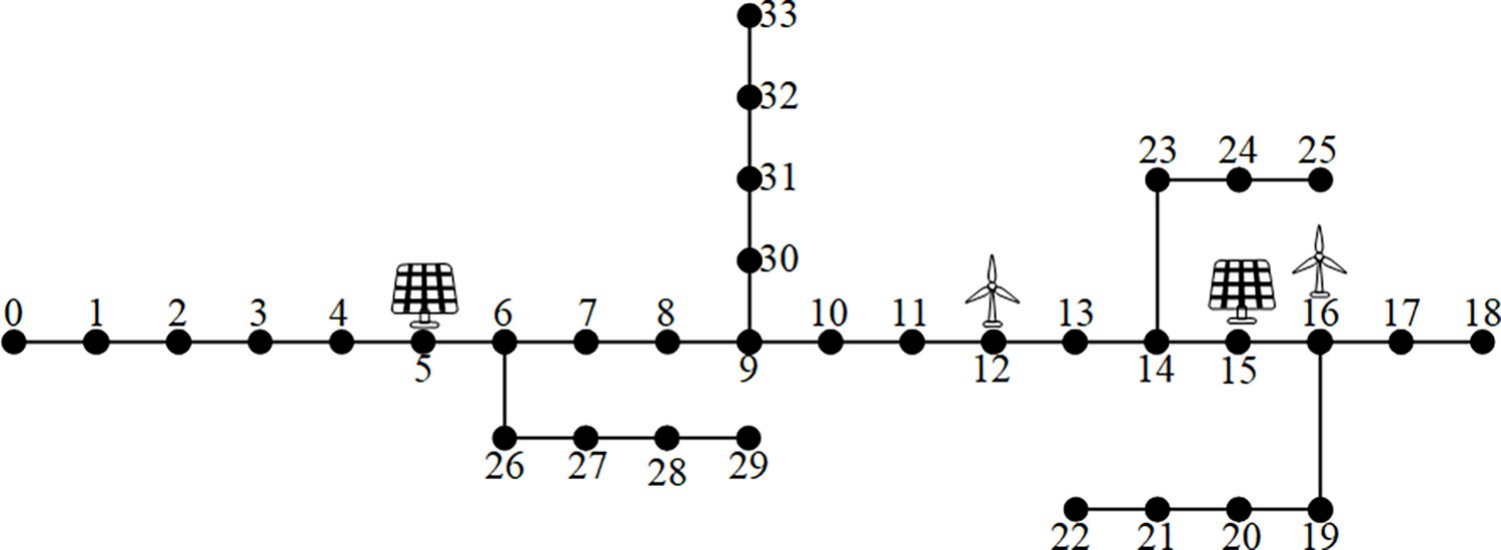
IEEE33 system topology.

To verify the effectiveness of the above processing and prediction methods, this paper utilizes the IEEE33 node system model as an example for simulation, constructs the line loss dataset and carries out the line loss prediction analysis. At nodes 5, 12, 15 and 16, solar power generation and wind power generation have been respectively established, and the network topology is shown in Fig 5.

Take the reference voltage *U*_*B*_ = 12.66 kV and the reference capacity *S*_*B*_ = 600 MVA. This example shows that the reactive power compensation equipment has been set up at the new energy source, and the new energy generation equipment does not absorb reactive power from the grid. Set node 0 as the balanced node, the voltage is 1.05 p.u., and the standard deviation of load fluctuation at each node is 5%. Set node 5 and 15 to access the photovoltaic power supply, node 12 and 16 to access the wind turbine, and the relevant parameters are shown in Table 1. Set the total new energy access capacity as 25% of the baseline capacity.

**Table 1:** New energy parameters.

Photovoltaic generator		Wind-driven generator	
Parameter	Value	Parameter	Value
Rated power	64MW	Rated power	86MW
Light intensity	α = 0.68	wind speed	c = 7m/s
Beta distribution	β = 6.78	Weibull distribution	k = 2.84
Photoelectric conversion rate	13.44%	cut-in wind speed	3m/s
Photovoltaic array area	1000m^2^	Rated wind speed	11m/s
Maximum light intensity	1000W/m^2^	Cut-out wind speed	25m/s
Node properties	PV	Node properties	PQ

### 5.1. Correlation analysis of line loss influencing factors

Since the data set is from the line loss data of a distribution network in China, the quality of the original data is high. According to the standard practice, we carried out the traditional data cleaning and normalization processing on the data set, subsequently, successfully meeting the requirements of the subsequent prediction process.

**Fig 6:**
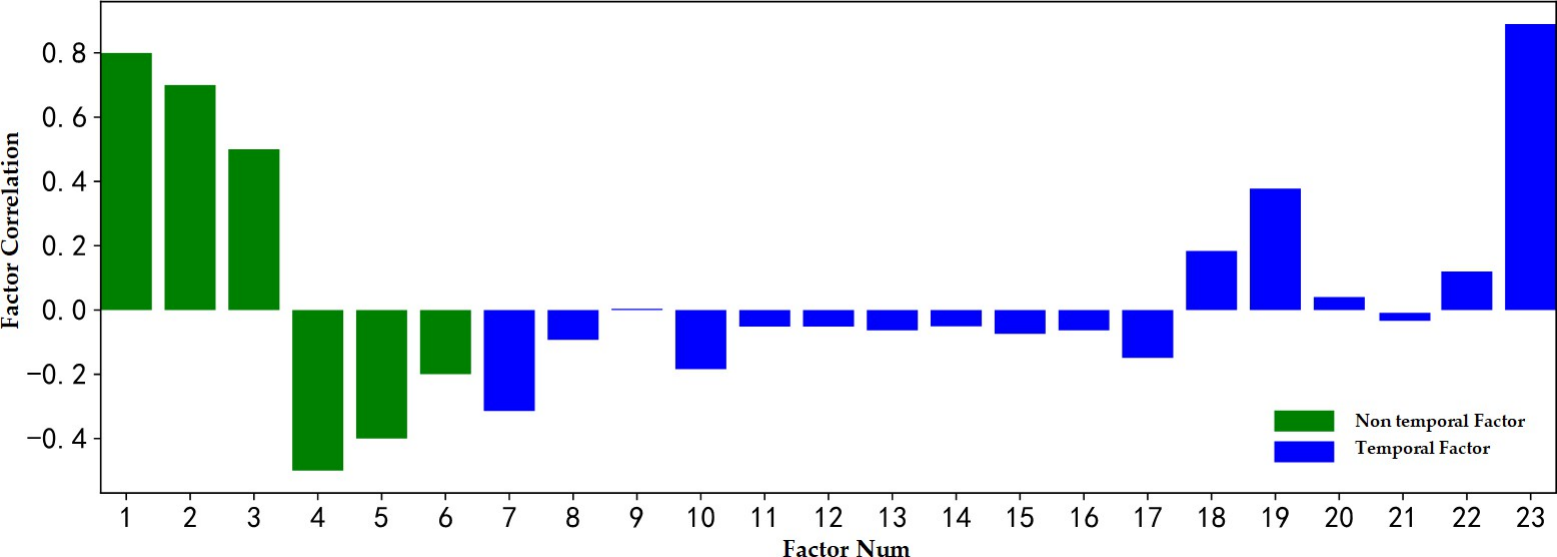
Correlation between each influencing factor and line loss.

Seventeen basic influencing factors related to timing and non-timing that affect the line loss are selected by manual experience, as shown in Table 2. For the non-timing influencing factors, the discrete processing form of solo thermal coding is used; for the timing influencing factors, the RNN recurrent neural network is used as a nonlinear function to recognize the importance of each timing influencing factor according to the idea of NARMA, and the corresponding hyper-parameter settings of the RNN recurrent neural network are shown in Table 3. The network weights are extracted to obtain the correlation ranking of the input influencing factor data, and the results are shown in Fig 6.

**Table 2:** Characteristic forms and numbers.

temporality	factor	continuity	Num	temporality	factor	continuity	Num
Nontemporal	Line resistance	dispersed	1	Temporal	Total radiation	continuous	13
	Line length	dispersed	2		Fixed radiation	continuous	14
	Transformer capacity	dispersed	3		Tracking radiation	continuous	15
	Transformer efficiency	dispersed	4		Photovoltaic power	continuous	16
	Reactive power compensation	dispersed	5		Precipitation	continuous	17
	Proportion of new energy	dispersed	6		Humidity	continuous	18
Temporal	Temperature	continuous	7		Surface pressure	continuous	19
	Azimuth	continuous	8		10m wind direction	continuous	20
	Cloud opacity	continuous	9		10m wind speed	continuous	21
	Dew point temperature	continuous	10		Zenith angle	continuous	22
	Scatter index	continuous	11		Grid load	continuous	23
	Direct index	continuous	12				

**Table 3:** NARMA hyperparameters.

hyperparameters	N	hyperparameters	N
Network	RNN	Epoch	1000
Input channels	17	Optimizer	Adam
Output channels	17	Loss function	MSE
Nonlinear activation function	tanh	Learning rate	0.01
Number of fully connected layer neurons	17		

From Fig 6, it is evident that among the six non-time series factors related to line loss, the line loss is significantly affected by the line resistance and length. Conversely, the total capacity of the transformer, transformer efficiency, capacity of the reactive power compensation device, and the ratio of new energy generation have a relatively smaller impact on line loss. Furthermore, the transformer efficiency, capacity of the reactive power compensation device, and the ratio of new energy generation are negatively correlated with the line loss. Among the 17 time series factors affecting line loss, the correlation coefficient between grid load and line loss is the highest, indicating the most intimate relationship between the two. Additionally, several other factors show a considerable correlation with line loss, such as temperature, dew point temperature, atmospheric precipitation, relative humidity, and surface air pressure. The correlations of other factors not involved in subsequent calculations are all less than 10%.

### 5.2. Line loss prediction and analysis with simulation modeling

#### 5.2.1. Specific realization of line loss prediction

To extract and separate the features of base loss and fast disturbance loss contained in the network-wide line loss data, the wavelet transform is applied to decompose the original line loss data into low-frequency components and high-frequency components. The Discrete Meyer wavelet, which is characterized by both smoothness and symmetry, is used to reduce edge distortion while maintaining signal continuity. At the same time, the scale function and the transformation function of the DMey wavelet are orthogonal in the frequency domain, which makes it possible to avoid introducing additional noise or distortions when reconstructing the signal.

At the physical level, the low-frequency components reflect the overall trend and slowly changing components of the line loss signal. It corresponds to the base loss of the line, the slowly transforming part of the loss caused by the change of the load portion; the high-frequency component reflects the spikes, sudden changes and other components of the line loss signal. It is mainly reflected in the high-frequency physical processes such as harmonics, shocks and transients in the line under the high proportion of new energy access.

**Fig 7:**
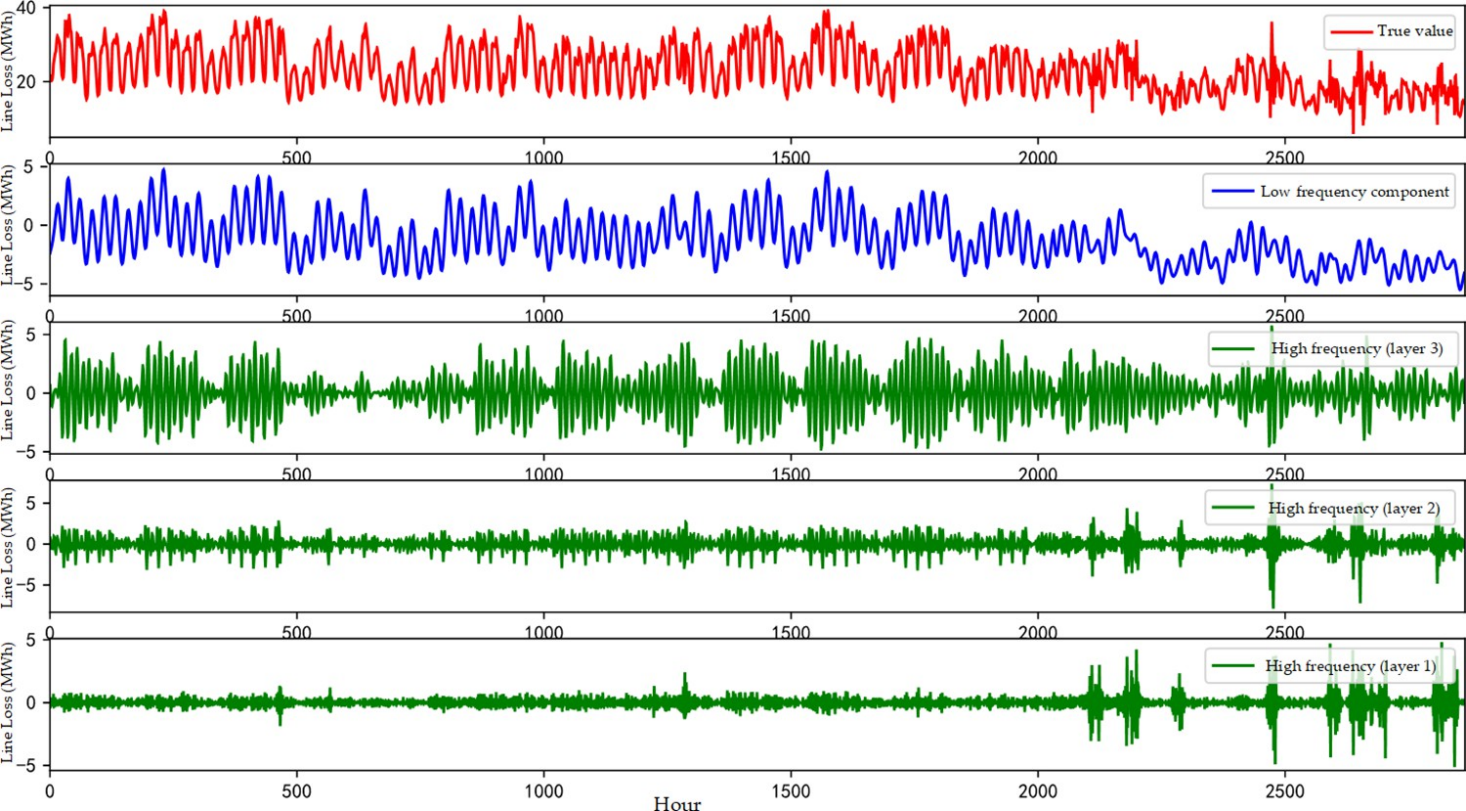
Signal map after wavelet transform.

Based on the above theory, the original network-wide line loss data within 4 months is analyzed by adopting a three-layer wavelet transform based on the DMey wavelet. The low-frequency signal and high-frequency signal of the line loss data are constructed, as shown in Fig 7.

As can be seen in Fig 7, the low-frequency component of the line loss data has a similar trend to the original line loss data, but the high-frequency characteristic waveform is significantly different from it. The high-frequency component waveform is similar to the pulse wave, and the high-frequency wave at the upper level has more significant volatility in the decomposition, reflecting the obvious volatility characteristics of the line loss data, which should be considered for modeling and prediction separately.

The dual-channel line loss prediction model for frequency domain specialization is established, and the inputs of the model use the key influencing factors and the high and low frequency components, the ratio of the training set to the test set is 8:2, and the parameters of the BIGRU-LSTM model are shown in Table 4. The Gaussian kernel function is used for Gaussian kernel density estimation with bandwidths of 0.95, 0.9, and 0.8, respectively. The prediction of the low-frequency components is achieved using deterministic prediction methods, whereas the quantitative modeling is performed using probabilistic prediction methods for the three-layer high-frequency components.

**Table 4:** BIGRU-LSTM model hyperparameters.

hyperparameters	N	hyperparameters	N
Input size	6	Batch as the first dimension	True
Output size	16	Number of fully connected layer neurons	32
Number of layers	1	Epoch	100
LSTM layers	1	Optimizer	Adam
LSTM hidden layer neurons	32	Loss function	MSE
Input window size	32	Learning rate	0.05

In deep learning, the hyperparameter is also an important factor affecting the accuracy of the model. In order to properly select the hyperparameter of the prediction model proposed in this paper, the value of the hyperparameter is selected according to the value of the loss function and the previous work experience [24]. In the control experiment, the selection of the super parameters of the control model is consistent with that of the model proposed in this paper, which proves that the prediction model proposed in this paper has higher accuracy under the same conditions.

**Fig 8:**
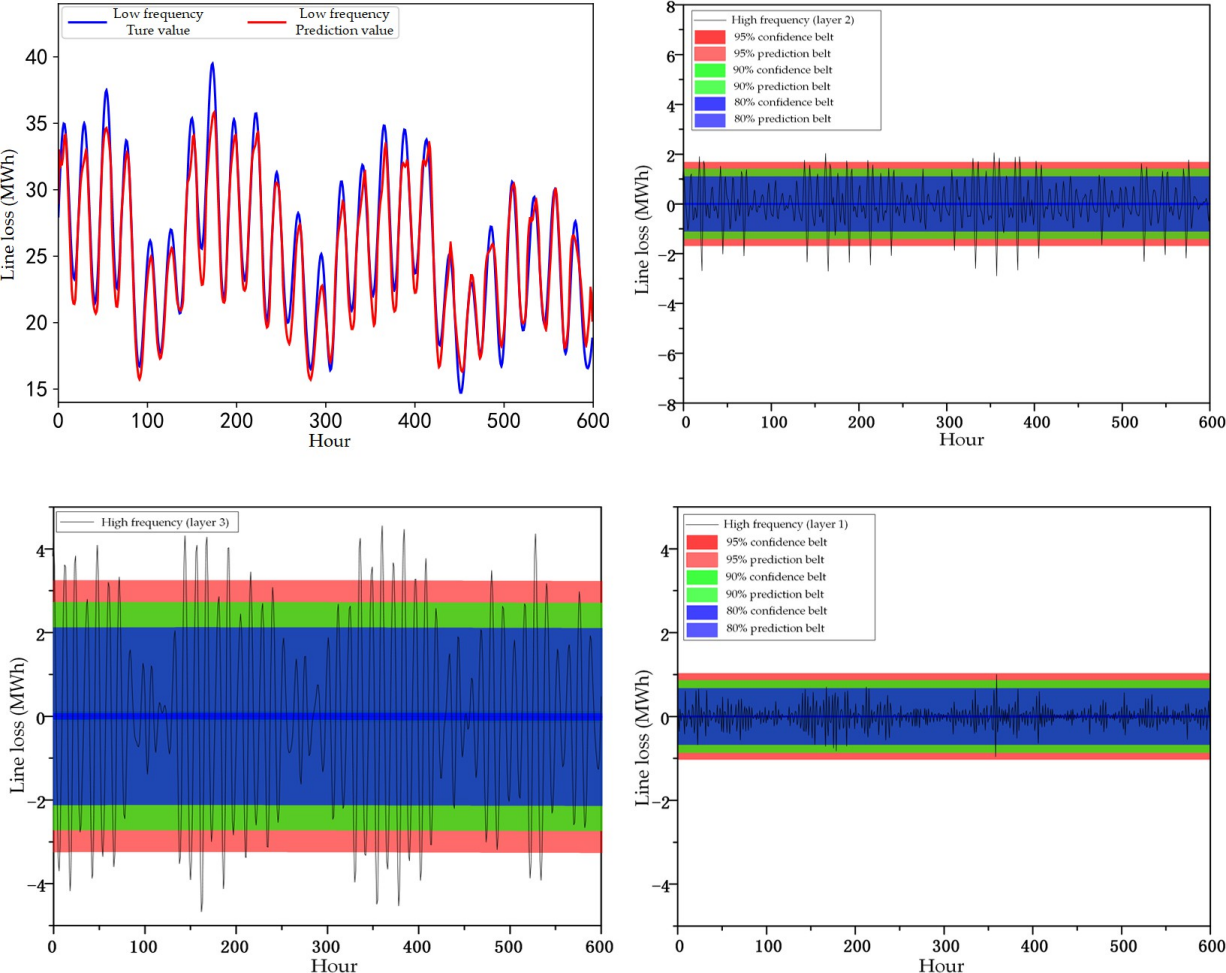
Line loss component prediction results. a) Low-frequency component (layer 3) model prediction results. b) Interval prediction results for high-frequency components (layer 3). c) Interval prediction results for high-frequency components (layer 2). d) Interval prediction results for high-frequency components (layer 1).

The predicted low frequency line loss and high frequency line loss prediction interval results for 25 days are obtained from the BIGRU-LSTM model and are shown in Fig 8.

In this paper, a three-layer wavelet transform decomposition is performed to predict the low-frequency third layer, high-frequency third layer, high-frequency second layer, and high-frequency first layer components, respectively, and then the prediction results of the four components are reconstructed and superimposed to obtain the total line loss prediction results. Thus the prediction results of the four line loss decomposition components are shown in Fig 8(A)–8(D). As can be seen from Fig 8, the results predicted by the low-frequency components have a higher degree of overlap with the actual data, and the overall effect of prediction is the better peak. For the high-frequency component prediction results, the prediction model given in this paper can give different probabilistic prediction intervals according to the change of high-frequency line loss, and the upper and lower bounds of the prediction intervals can cover the change interval of high-frequency components of line loss.

**Fig 9:**
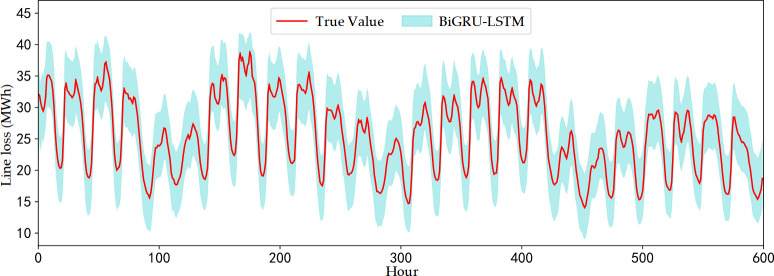
Crossover prediction superimposed on reconstruction prediction results.

Further, the upper and lower bounds of the prediction intervals of the high-frequency components and the deterministic prediction results of the low-frequency components are superimposed and reconstructed by using the inverse wavelet transform, and the probabilistic prediction results of the line loss of the high-ratio new energy distribution network are obtained, as shown in Fig 9.

As can be seen in Fig 9, the prediction interval given by the model can cover the range of changes in the theoretical line loss curve, which correctly reflects the trend of line loss data. At the same time, the model will adjust the range of prediction intervals according to the uncertainty of line loss changes to adapt to the dynamic distribution characteristics of line loss, i.e., for the part of the rising and falling process of the line loss, only a narrower prediction interval is needed to cover the expected changes; however, it is necessary to expand the range of intervals to cover the fluctuating range of the line loss in the peaks and valleys of the line loss time series. It shows that the nonparametric intervals constructed by the line loss prediction modeling framework proposed in this paper can flexibly adjust with the trend of line loss changes, and have the adaptability to adapt well to the fluctuating line loss prediction.

#### 5.2.2. Line loss prediction performance analysis

**Fig 10:**
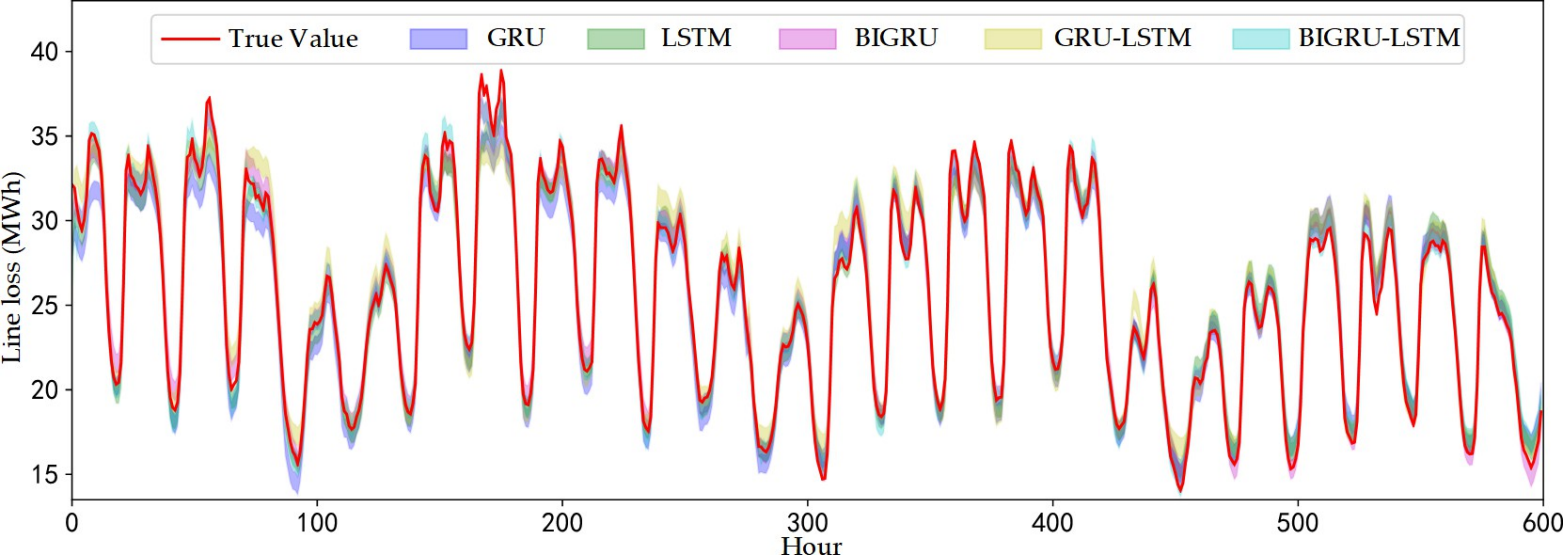
Control chart of the ablation experiment.

To analyze the contribution of model components to the overall model performance, ablation experiments are used for comparative analysis. In this paper, four algorithmic models, GRU, BIGRU, LSTM, and GRU-LSTM, are used in conjunction with kernel density estimation, the same prediction and superposition reconstruction methods are used to conduct ablation experiments and carry out a comparative analysis of the results. A relatively low confidence level of 30% is selected to obtain more discriminative evaluation results, which is because the prediction intervals are narrower at lower confidence levels, requiring higher prediction accuracy of the models. The hyperparameter settings for each algorithm are shown in Table 5. The results of the comparison of line loss prediction intervals for the selected 25 days are shown in Fig 10.

**Table 5:** Hyperparameters for each comparison model.

comparison model	Hyperparameters
GRU	Input size:6, hidden layers:6
BIGRU	Input size:6, hidden layers:6
LSTM	Input size:6, hidden layers:6
GRU-LSTM	Input size:6, hidden layers:32

As can be seen in Fig 10, the five models corresponding to the banded areas have a high degree of overlap, and several models have corresponding advantages in predicting the trend of line loss, and the prediction results can better match the actual value of distribution network line loss. Four deterministic evaluation indexes, MAE, MAPE, RMSE and R2, and two probabilistic evaluation indexes are used for specific assessment of prediction performance, and the results are shown in Table 6.

**Table 6:** Predictive performance analysis of ablation experiments.

Prediction Model	MAE	MAPE	RMSE	R2	*δ*_cov_	*χ*_cov_
GRU	0.868	3.382	1.098	0.965	0.357	1.094
BIGRU	0.483	1.928	0.609	0.989	0.5	1.115
LSTM	0.648	2.546	0.868	0.978	0.673	1.145
GRU-LSTM	0.918	3.586	1.195	0.958	0.395	1.140
BIGRU-LSTM	0.410	1.725	0.576	0.990	0.758	1.151

From the ablation experiment results in Table 6, it can be seen that compared to unidirectional GRU, BIGRU performs better in all evaluation metrics, e.g., the MAPE reduces by 0.43 and the RMSE reduces by 0.45. This is mainly attributed to the fact that bidirectional RNNs can synthesize past and future contextual information for sequence modeling. Meanwhile, the model GRU-LSTM, which integrates GRU and LSTM, also outperforms the single GRU, proving that the integrated model can improve the robustness of line loss prediction.

According to the MAPE, R2, and *δ*_*cov*_ indicators of the BIGRU-LSTM model, the model shows the best line loss prediction effect both in terms of deterministic and probabilistic indicators, which fully proves the effectiveness of the bidirectional structure and integration strategy in this paper, and achieves a better effect of the complementary synergy of the two structures. In addition to the improved prediction accuracy, the prediction interval coverage of BIGRU-LSTM reaches 0.758, which is also significantly higher than that of other comparative models, indicating that the prediction intervals output from BIGRU-LSTM is more reliable, which ensures the robustness of the time series prediction of line loss.

### 5.3. Example line loss prediction analysis

In the prediction study in sections 5.1–5.2, the selected parts are the low-frequency part of the line loss curve that changes relatively steadily for prediction and analysis, but in fact, in the distribution network the line loss value may change drastically within a short period of time, for example, the load change (switching of factories, starting/stopping of large-scale equipment, demand for electricity at different times in the residential area, etc.), the change of the grid configuration (switching of lines, starting/stopping of transformers, etc.), power supply change (starting/stopping of power stations, change of renewable energy supply, etc.), or the change of weather conditions, equipment failure, etc. (line switching, starting/stopping of transformers, etc.), changes in power sources (starting/stopping of power stations, changes in the amount of electricity supplied by renewable energy sources, etc.), or changes in weather conditions, equipment failures, etc. If deterministic line loss prediction methods are used for the high-frequency part of the prediction will lead to serious prediction distortion. In this paper, the high-frequency component of the strongly volatile part of the line loss is predicted using a probabilistic prediction method and analyzed as follows.

**Fig 11:**
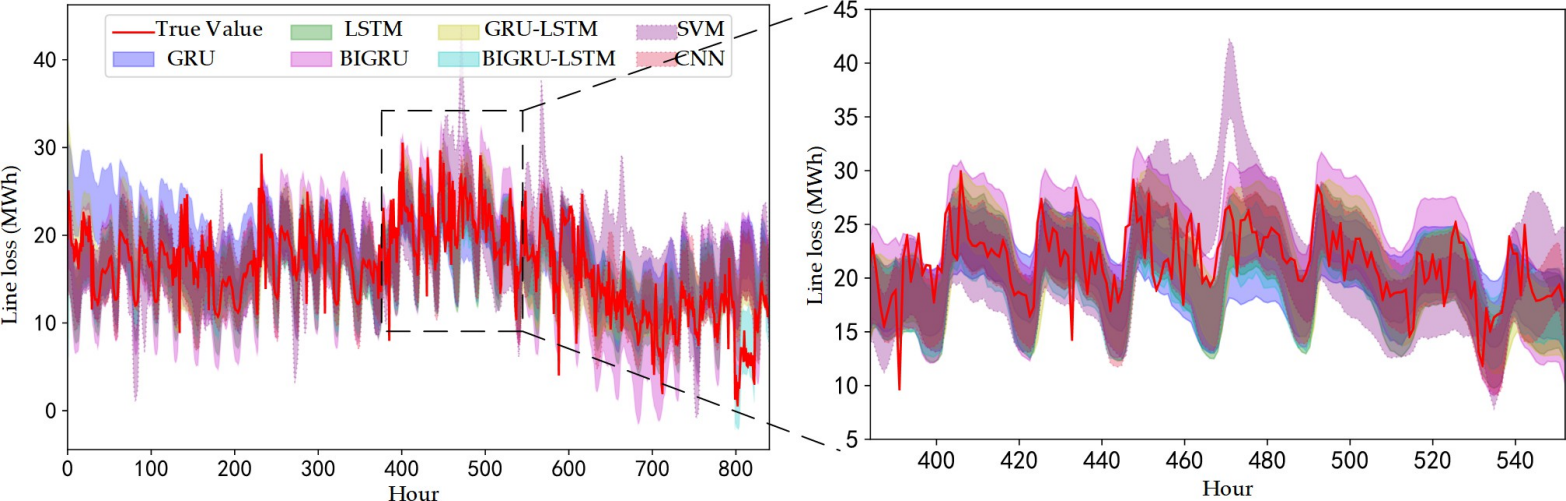
Prediction results of example line loss. (a) Prediction curve for instance line loss. (b) Localized result curve for days 17–23.

Based on the time series data of line loss for a certain month and the next month of a local power grid in Norway for a total of 840 hours in the approaching 5 weeks (35 days), several prediction models are selected to make probabilistic prediction of line loss for the power grid at 90% confidence level, and the results of the comparison of prediction intervals are shown in Fig 11.

As can be seen in Fig 11(A), the theoretical curve of line loss shows strong volatility, and the prediction effect of each model on the theoretical curve varies. Meanwhile, in addition to the overall strong present volatility of the theoretical curve, the curve possesses stronger volatility characteristics in some intervals, such as the 17th - 23rd day (corresponding to the 384th - 552nd hour of the horizontal coordinate) is more volatile compared to other periods, as shown in Fig 11(B).

To determine the prediction effect of the BIGRU-LSTM model in Fig 11(A), based on GRU, BIGRU, LSTM, and GRU-LSTM, the traditional models SVM and CNN models are added for comprehensive comparison, and the results of the prediction performance indexes are compared as shown in Table 7. Among them, the weights of C and are taken as 0.7 and 0.3, respectively.

**Table 7:** Example monthly line loss prediction performance comparison.

Prediction Model	MAE	MAPE	RMSE	R2	*δ*_cov_	*χ*_cov_	*Score*
GRU	2.498	13.068	3.101	0.760	0.578	6.766	0.272
BIGRU	2.281	9.080	2.868	0.794	0.809	7.439	0.444
LSTM	2.905	12.726	3.697	0.658	0.729	9.681	0.308
GRU-LSTM	2.638	10.937	3.364	0.765	0.748	6.936	0.408
SVM	1.946	10.916	2.513	0.639	0.644	7.909	0.292
CNN	1.482	9.708	2.332	0.673	0.779	7.014	0.432
BIGRU-LSTM	1.717	7.021	2.131	0.886	0.84	7.223	0.476

As seen in Table 7, the MAPE of BIGRU-LSTM is significantly lower than other models, and R2 is significantly higher than other models. As a result, from the deterministic assessment index, the line loss prediction based on the BIGRU-LSTM model in this paper is better than other comparative methods. From the probabilistic assessment index, BIGRU-LSTM also shows high probability prediction accuracy different from other models, and its prediction interval coverage indicates that the probability of the future line loss value appearing in the prediction interval predicted by BIGRU-LSTM is the largest. The width of the prediction interval at this point is in the upper middle range compared to other models, indicating that the fluctuations in line losses are so large that it is difficult to encompass major line loss variations in a smaller interval. From the analysis of the combined weighted scores, the BIGRU-LSTM obtained the largest score value as well, indicating that this model can be applied to the monthly prediction of line loss.

To further illustrate the effectiveness of the probabilistic prediction method proposed in this paper for the application of localized strong fluctuation line loss prediction, the predicted line loss data for days 17–23 (corresponding to hours 384–552 in the horizontal coordinate) shown in Fig 11(B) are used as an example for the performance evaluation, and the comparison results are shown in Table 8. This week, the new energy connected to this grid evaluates the largest generation capacity and produces the largest relative line loss volatility.

**Table 8:** Example weekly line loss prediction performance comparison.

Prediction Model	MAE	MAPE	RMSE	*δ*_cov_	*χ*_cov_	*Score*
GRU	2.575	9.469	3.376	0.518	5.089	0.320
BIGRU	2.530	9.878	3.178	0.649	7.195	0.373
LSTM	2.764	10.533	3.448	0.725	8.559	0.398
GRU-LSTM	2.929	12.283	3.713	0.673	7.902	0.371
SVM	3.738	12.073	4.757	0.595	5.509	0.380
CNN	2.578	9.720	3.284	0.702	7.677	0.407
BIGRU-LSTM	2.618	9.691	3.294	0.727	6.849	0.460

According to the prediction interval coverage, although BIGRU-LSTM is still relatively good, the coverage of LSTM is similar to it, and only using the prediction interval coverage is not enough to indicate which model has excellent performance. By analyzing the prediction interval widths and the combined weighted scores, BIGRU-LSTM has smaller and higher prediction interval widths with the same prediction interval coverage. It can be shown that BIGRU-LSTM is also better in weekly prediction. However, due to the problem of close prediction interval coverage, the advantage of the model is not prominent in weekly prediction. This is because when training the model, the model adaptively optimizes the tuning parameter for the law of a 35-day time scale, while the model suffers from under-optimization of the detail parameters when only a part of the time period is considered. In subsequent studies, both monthly prediction error and weekly prediction error can be used as the joint optimization objective during model training, so that the model can achieve better results in both monthly and weekly prediction.

With the support of enough prediction networks and data samples, the prediction results of comparative experiments show that the proposed bigru-lstm prediction architecture has the best prediction performance. In addition, the ablation experiment verified the advantages of BIGRU-LSTM structure compared with other traditional model single structure, and the performance of each evaluation index was more excellent. Finally, in order to verify the robustness of BIGRU-LSTM, the line loss data of a Norwegian power grid is introduced for example analysis. The results show that the prediction model proposed in this paper still performs best on the data set with strong volatility, so it is concluded that the prediction model has high prediction accuracy and strong adaptability.

## 6. Conclusions

This paper proposes a new method of frequency division line loss prediction for high proportion new energy distribution network based on BIGRU-LSTM model. The main works of this prediction method are:

1. After data cleaning and related data preprocessing of the original data set, a line loss data set based on the power grid of a region in China is established, including the corresponding data set of temporal factors and non-temporal factors, and is divided into the training set and the verification set according to the ratio of 8 to 2.
2. Using the classical grey correlation analysis and the improved NARMA method, the weight coefficients of the influence factors of time series and non-time series are calculated accurately, and the feature data set is further reduced and processed accurately.
3. Wavelet transform is used to decompose the original line loss data to better capture the high frequency part of rapid fluctuation and the relatively stable low fre-quency part of the line loss historical data, which improves the multi-scale analysis capability of the prediction model and provides a basis for subsequent dual channel prediction.
4. BIGRU-LSTM model cooperates with wavelet transform to realize frequency division probability prediction. The dual channel prediction model is used to train and predict the line loss data of each frequency band, and the final prediction results are obtained by integrating the sequences of each frequency band. This frequency division prediction framework reduces the complexity of the model, and takes into account the accuracy of trend prediction and the rationality of volatility prediction.

Outlook: This paper does not currently include the error correction module, but our future research will focus on this area. We plan to explore how error feedback information can optimize the training process of LSTM models through backpropagation mechanisms. This will involve tracking errors between model outputs and actual observations during training, and adjusting network weights and the on-off states of gated units based on these errors to enhance the model’s modeling power and predictive accuracy for complex time series. By enhancing our proposed BIGRU-LSTM model, we aim to improve its performance in line loss prediction. Through this enhancement measure, the line loss prediction model can provide valuable insights for reducing losses in advance, optimizing power grid operations, and implementing measures such as regulating power supply modes, adjusting equipment layouts, and reducing losses based on accurate line loss prediction results.

Due to limited data sets and space, no further verification of the prediction method in this paper has been carried out for distribution network line losses with different access locations and capacities. For the scalable new energy access system, the method in this paper can keep the weight of the underlying network unchanged, and only fine tune the output layer or the top weight to adapt to the new distribution network structure changes, without starting from scratch training, so that the prediction model proposed in this paper can obtain a certain migration learning ability, improve the adaptability and efficiency of the neural network, which is the next step to focus on.
